# Ultrasound-guided angioplasty of arteriovenous fistula for hemodialysis: initial experience of a single center

**DOI:** 10.1590/1677-5449.202600222

**Published:** 2026-09-03

**Authors:** Claudia Guimarães Agle, Fábio Luis Barbosa Andrade, Ana Clarice de Carvalho Bacelar, Giovana Pires de Sousa Bonfim, Eduardo Castro de Oliveira, José Siqueira de Araujo

**Affiliations:** 1 Hospital Ana Nery – HAN, Salvador, BA, Brasil.; 2 Complexo Hupes – UFBA, Salvador, BA, Brasil.

**Keywords:** arteriovenous fistula, angioplasty, Doppler ultrasonography, hemodialysis, vascular access

## Abstract

Chronic Kidney Disease is a growing public health concern and arteriovenous fistulas (AVFs) are the preferred type of vascular access for hemodialysis, but venous stenosis can impair their function. Angioplasty is the first-line treatment, but availability is limited on the public health care system. This retrospective study evaluated the feasibility and early outcomes of ultrasound-guided AVF angioplasty performed at a referral center in 2024. Nine patients were recruited and followed for 3 months. Outcomes included technical success, blood flow, venous diameter, complications, and patency. Technical success was achieved in 77.8% of cases, with increased blood flow and venous diameter. Complications occurred in 66.6%, the majority minor hematomas, although two patients developed acute thrombosis. The technique was feasible and improved hemodynamic parameters, although with a non-negligible complication rate.

## INTRODUCTION

Chronic kidney disease (CKD) is a significant public health problem, with a growing impact on health care systems. Data from the 2022 Brazilian Dialysis Census indicate that there are more than 150 thousand patients on dialysis in the country, with increased use of central venous catheters and a drop in the rate of arteriovenous fistula (AVF) usage, demonstrating problems related to creation, maturation, and maintenance of AVFs.^1^

The ideal vascular access for hemodialysis should offer adequate flow rate, a low rate of complications, and lasting patency. In view of these criteria, AVFs remain the best option and are associated with lower rates of morbidity and mortality than central venous catheters.^2^

Notwithstanding, AVFs often develop venous stenosis secondary to intimal hyperplasia, compromising maturation and functionality.^3^ Brazilian guidelines recommend conducting Doppler ultrasound if cannulation fails after 12 weeks, to identify problems and plan treatment.^4^

Percutaneous transluminal angioplasty is considered the treatment of choice in the majority of cases. However, the conventional method requires highly complex infrastructure and use of iodinated contrast and ionizing radiation, which restricts its availability on Brazil’s Unified Health System (SUS - Sistema Único de Saúde). In this context, ultrasound-guided angioplasty has emerged as a promising alternative option, enabling less expensive interventions, without exposure to radiation or contrast, and is especially useful in pre-dialytic patients or those with contraindications to iodinated contrast.

## OBJECTIVES

Primary objective: to assess the feasibility of ultrasound-guided angioplasty of arteriovenous fistulas

Secondary: to describe variables associated with the procedure’s feasibility, including blood flow before and after, complications, and short term patency.

## METHODS

A retrospective, observational, cohort study was conducted in accordance with the Strengthening the Reporting of Observational Studies in Epidemiology (STROBE) guidelines.^5^ The study recruited all patients who underwent ultrasound-guided AFV angioplasty during 2024, assembling a convenience sample.

The inclusion criteria were patients over the age of 18 years, with an AVF that had developed significant stenosis (> 70%) linked to restricted flow (< 600 mL/min) (Figure 1) or failure to mature. There were no exclusion criteria.

**Figure 1:**
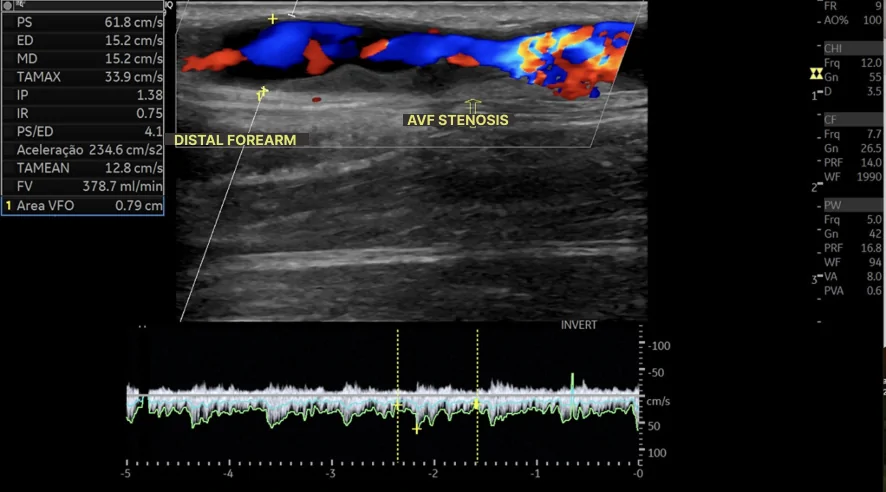
Stenosis of an AVF seen on Doppler ultrasound, with turbulent blood flow. AVF = arteriovenous fistula.

Data on blood flow and venous diameter were collected before and after the procedure (Figures 2 and 3), and complications (Figure 4) and ultrasonographic assessment findings at 3 months were also analyzed.

**Figure 2:**
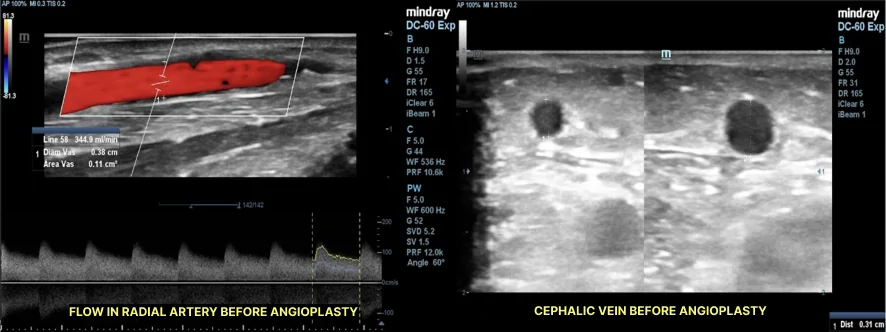
Blood flow in the radial artery (before angioplasty) + diameter of the cephalic vein (before angioplasty).

**Figure 3:**
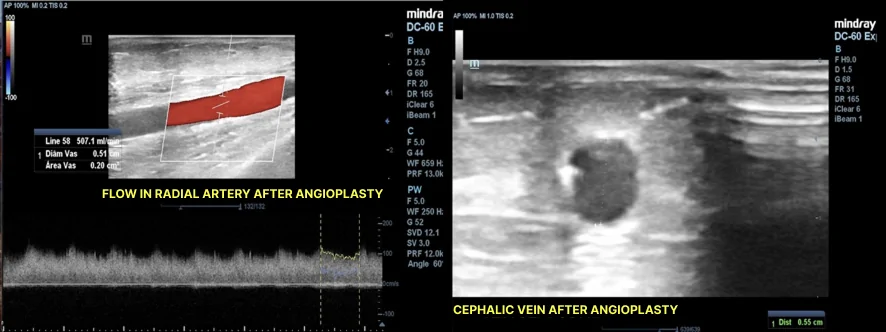
Blood flow in the radial artery (after angioplasty) + diameter of the cephalic vein (after angioplasty).

**Figure 4:**
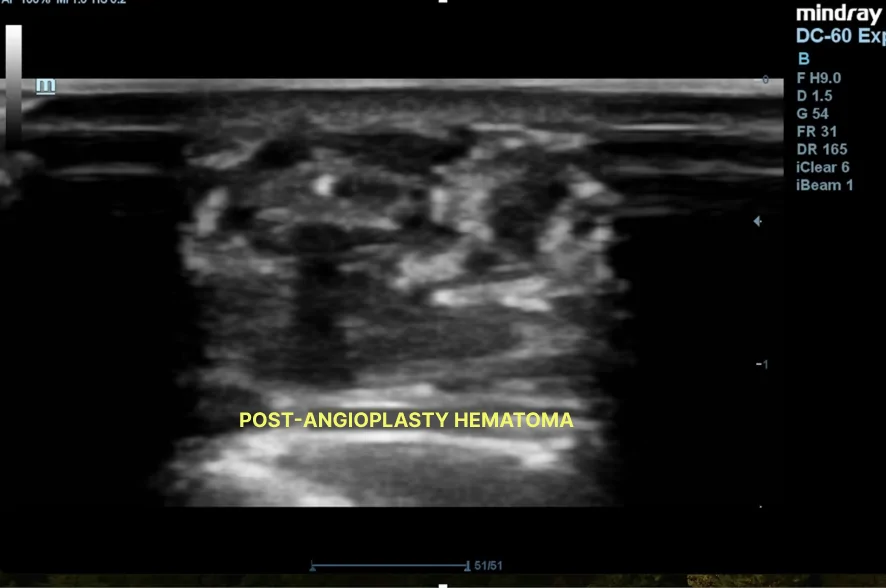
Post-angioplasty hematoma seen on Doppler ultrasound.

Technical success was defined as restoration of blood flow and recanalization of stenosis with immediate patency at the end of the procedure. Patency was defined as maintenance of AVF function at 3-month follow-up, with no need for reintervention and no evidence of thrombosis on Doppler ultrasound.

The research project was submitted to and approved by the Research Ethics Committee at the Hospital Ana Nery (HAN), in Salvador, Bahia, Brazil, and registered on the Plataforma Brasil (Ethics Appraisal Submission Certificate number 83561224.1.0000.0045 and Substantiated Opinion number 7.511.150).

### Statistical analysis

For statistical analysis, data were expressed as mean and standard deviation. Values from before and after the procedure were compared using Wilcoxon’s nonparametric test for paired samples, considering a significance level of 5%. The analysis was exploratory, because of the small sample size.

### Angioplasty technique and materials

Procedures were performed with the aid of Doppler ultrasound. Balloons were chosen according to technical criteria and availability at the institution.

1. Procedures were performed using a MindRay Doppler ultrasound machine. First, Doppler ultrasound mapping was conducted to identify the site and degree of stenosis.
2. Sedation + anesthetic nerve block.
3. Retrograde ultrasound-guided puncture of the radial artery to obtain percutaneous access, followed by introduction of a guidewire under ultrasonographic guidance. A 5Fr radial introducer, 0.035 x 260 mm hydrophilic guidewire, and vertebral catheter were employed. Under ultrasound guidance, the guidewire was advanced through the stenosed segment, with real-time monitoring.
4. A balloon catheter was positioned over the guidewire into the center of the area of stenosis. Dilatation was performed with ultrasound control, observing the balloon expand (Figure 5). Conquest and Mustang balloons sized from 5 to 7 mm were used (Table 1 lists the characteristics and sizes).

**Figure 5:**
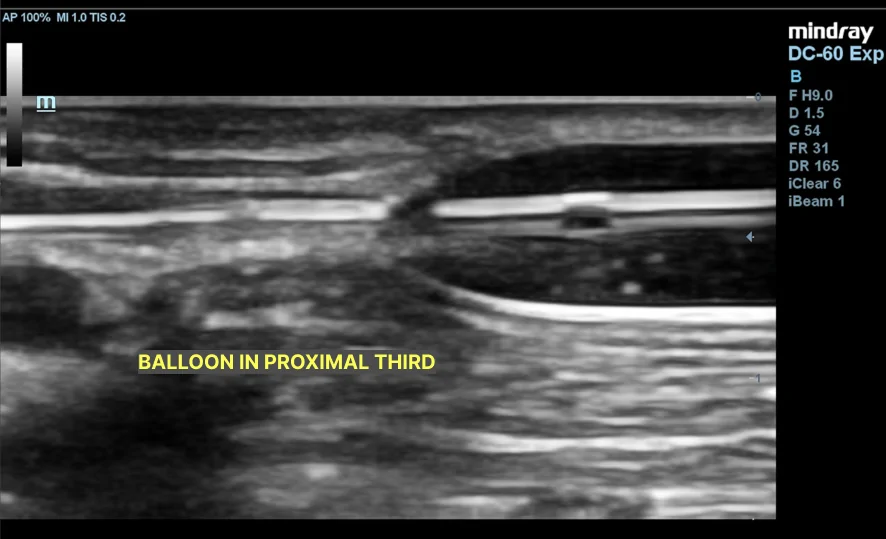
Ballooning of stenosis seen on Doppler ultrasound.

**Table 1:** Technical characteristics of the balloons used in the angioplasty procedures.

	**Diameter/ Length**	**Compliance/pressure**	**Brand**
Patient 01	5x60 mm	Non-compliant	Conquest – BD/Bard
	6x60 mm	Ultra high pressure	
Patient 02	5x40 mm	Non-compliant	Conquest – BD/Bard
	7x40 mm	Ultra high pressure	
Patient 03	5x40 mm	Non-compliant	Conquest – BD/Bard
	7x40 mm	Ultra high pressure	
Patient 04	6x60 mm	Non-compliant	Conquest – BD/Bard
		Ultra high pressure	
Patient 05	5x80 mm	Non-compliant	Conquest – BD/Bard
	7x80 mm	Ultra high pressure	
Patient 06	Thrombosis during catheterization	-	-
Patient 07	5x40 mm	Semi-compliant	Mustang - Boston
	6x40 mm	High pressure	
Patient 08	6x60 mm	Semi-compliant	Mustang - Boston
	7x60 mm	High pressure	
Patient 09	6x40 mm	Semi-compliant	Mustang - Boston
	7x40 mm	High pressure
5. After dilatation, ultrasound was used to check that stenosis had been resolved and flow had increased and to rule out immediate complications.
6. The introducer was removed and local compression applied.

## RESULTS

A total of nine ultrasound-guided AVF angioplasty procedures were performed and their respective variables were assessed (diameter and blood flow) (Tables 2, 3, and 4). Technical success was achieved in seven cases (77.8%), but two patients (22.2%) developed acute thrombosis of the fistula during the procedure, constituting treatment failure.

**Table 2:** Characteristics of patients who underwent ultrasound-guided AVF angioplasty (n = 9).

**Variable**	**Result**
Male, n (%)	6 (66.7)
Female, n (%)	3 (33.3)
Radiocephalic AVF, n (%)	6 (66.7)
Brachiocephalic AVF, n (%)	3 (33.3)
On dialysis, n (%)	6 (66.7)
Pre-dialysis, n (%)	3 (33.3)
Technical success, n (%)	7 (77.8)
Technical failure (occlusion), n (%)	2 (22.2)
Type of balloon, n (%)	
Non-compliant (Conquest)	6 (66.7)
Semi-compliant (Mustang)	3 (33.3)

AVF = arteriovenous fistula.

**Table 3:** Blood flow through AVF before and after ultrasound-guided angioplasty (ml/min).

	**AVF blood flow before**	**AVF blood flow after**	**Type of AVF**
Patient 01	70.0 ml/min	478.0 ml/min	Radiocephalic
Patient 02	300.0 ml/min	850.0 ml/min	Radiocephalic
Patient 03	234.0 ml/min	Acute AVF occlusion	Radiocephalic
Patient 04	190.0 ml/min	700.0 ml/min	Radiocephalic
Patient 05	152.0 ml/min	787.0 ml/min	Radiocephalic
Patient 06	253.4 ml/min	Acute AVF occlusion	Brachiocephalic
Patient 07	344.9 ml/min	507.1 ml/min	Radiocephalic
Patient 08	177.8 ml/min	515.9 ml/min	Brachiocephalic
Patient 09	346.6 ml/min	500.0 ml/min	Brachiocephalic

AVF = arteriovenous fistula.

**Table 4:** Diameter of AVF vein before and after ultrasound-guided angioplasty (mm).

	**Vein diameter before**	**Vein diameter after**
Patient 01	2.0 mm	6.0 mm
Patient 02	2.0 mm	6.0 mm
Patient 03	3.7 mm	AVF occluded
Patient 04	2.0 mm	5.6 mm
Patient 05	2.8 mm	5.8 mm
Patient 06	2.0 mm	AVF occluded
Patient 07	3.1 mm	5.5 mm
Patient 08	4.1 mm	4.3 mm
Patient 09	4.0 mm	4.0 mm

AVF = arteriovenous fistula.

The comparative analysis of hemodynamic parameters only included cases in which technical success had been achieved and data were complete (n = 7), excluding patients who had developed acute intraoperative thrombosis.

Mean blood flow increased from 225.9 ml/min before the procedures to 619.7 ml/min after the procedures (p = 0.018; Wilcoxon test). Mean vein diameter increased from 2.9 mm to 5.3 mm, with a statistically significant difference (p = 0.017) (Table 5).

**Table 5:** Statistical analysis of hemodynamic parameters (only includes cases with technical success, n = 7).

**Variable**	**Before (mean ± SD)**	**After (mean ± SD)**
Blood flow (ml/min)	225.9 ± 107.7	619.7 ± 154.8
Vein diameter (mm)	2.9 ± 0.9	5.3 ± 0.7

SD = standard deviation.

Complications occurred in 66.6% of cases, the majority of which were local hematoma (44.4%). Major complications occurred in 22.2% of cases, with two cases of acute fistula thrombosis (Table 6).

**Table 6:** Complications observed after ultrasound-guided AVF angioplasty– n (%).

Hematoma, n (%)	4 (44.4%)
Acute AVF occlusion, n (%)	2 (22.2%)

AVF = arteriovenous fistula.

The 3-month ultrasound assessments showed that patency was maintained in 77.8% of cases (seven of nine patients), i.e., in 100% of the cases in which immediate success had been achieved, with flow and vein diameter maintained in all functional cases (Tables 7 and 8; Figure 6).

**Table 7:** Ultrasonographic assessment of AVF 3 months after ultrasound-guided angioplasty.

	**Vein diameter after 3 months**	**AVF blood flow after 3 months**
Patient 01	6.0 mm	850.0 ml/min
Patient 02	7.0 mm	800.0 ml/min
Patient 03	Thrombosis	AVF occluded
Patient 04	5.9 mm	498.0 ml/min
Patient 05	9.1 mm	989.9 ml/min
Patient 06	Thrombosis	AVF occluded
Patient 07	6.0 mm	1200.0 ml/min
Patient 08	4.9 mm	560.0 ml/min
Patient 09	6.0 mm	690.0 ml/min

AVF = arteriovenous fistula.

**Table 8:** Ultrasonographic assessment at 3 months (successful cases, n = 7).

**Variable**	**Mean ± SD**
Blood flow (ml/min)	798.3 ± 238.7
Vein diameter (mm)	6.4 ± 1.3

SD = standard deviation. Patency at 3 months: 100% of cases with immediate technical success (cases with technical failure excluded from analysis).

**Figure 6:**
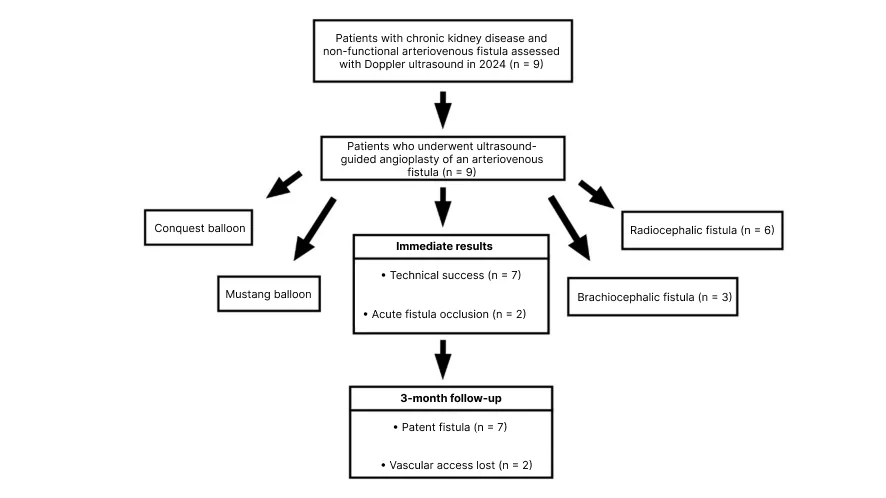
Flow diagram.

## DISCUSSION

Ultrasound-guided angioplasty of AVFs has been progressively incorporated as an alternative to conventional treatment, especially in scenarios with limited resources. In the present study, the procedure was technically feasible in the majority of cases, with a 77.8% success rate, associated with significant improvement of hemodynamic parameters.

In the cases in which technical success was achieved, blood flow and vein diameter increased, both of which are parameters with fundamental importance to fistula functionality. In agreement with reports in the literature, these findings indicate that flow measured with Doppler is a marker of clinical success after interventions in accesses for hemodialysis,^6,7^ and the values are close to those in the criteria described by the Kidney Disease Outcomes Quality Initiative (KDOQI) guidelines.^8^

Doppler ultrasound has a central role to play in AVF assessment, follow-up, intervention, because it is a widely-available, noninvasive method that can provide anatomic and functional information in real time.^6,7^

Ultrasound-guided angioplasty offers significant advantages in relation to the conventional method, including elimination of use of ionizing radiation and iodinated contrast, which is particularly important in patients with pre-dialytic chronic kidney disease. Additionally, the possibility of performing the procedure in settings without high complexity infrastructure widens access to treatment, particularly in health care systems with structural limitations.^3,6^

Previous studies reported favorable results using this technique. Abbadie et al.^6^ and Kazandjian et al.^7^ described improved hemodynamic parameters and adequate short-term patency. More recently, a Brazilian multicenter study with drug-eluting balloons reported good results for efficacy and safety, with medium-term patency.^9^ While the present study did not use drug-eluting balloons, the data provide additional support for the role of angioplasty in management of AVF dysfunctions.

Nonetheless, the results of the present study should be interpreted in the light of the complication rate observed. While the majority of the adverse events were minor complications, such as self-limiting hematoma, the overall rate was high (66.6%), including acute fistula thrombosis, in 22.2% of cases, causing failed procedures. Although most of these complications did not have any clinical impact on the function of the hemodialysis access, the frequency with which they were observed is not negligible and should be analyzed critically.

In the literature, DerDerian et al.^10^ describe non-negligible rates of complications, with predominance of less important events and a lower incidence of thrombosis. The elevated rate observed in this study may be related to the small sample size, the heterogeneity of the sample, and the operator-dependent technique.

Additionally, it should be considered that AVF stenosis is predominantly fibrotic, often demanding use of high-pressure balloons to achieve dilatation. This may contribute to a greater risk of vascular injury during the procedure, influencing the occurrence of complications.

Another relevant point is related to the different materials employed. Several different types and sizes of balloons were used in the cases analyzed, chosen in response to availability at the institution, which is a scenario that echoes the situation at many centers, but one that may affect the results, especially in more complex injuries.

Despite these limitations, ultrasound-guided angioplasty can constitute a feasible alternative option for treatment of AVF stenosis and failure to mature, especially in settings with limited access to cath labs, as pointed out in recent guidelines.^4,11,12^

This study has limitations. The sample is small and was selected by convenience, which limits its statistical power and hinders generalization of the findings. Additionally, the sample was heterogeneous, including both patients on dialysis and patients not receiving dialysis, in addition to different types of AVF. The range of different materials used, a result of varying availability at the institution, and the operator-dependent procedure may have influenced the outcomes observed. Finally, the rate of complications, including major events such as acute fistula thrombosis, should be interpreted with caution, especially considering the small sample size.

## CONCLUSIONS

Ultrasound-guided AVF angioplasty was feasible in selected cases, with improvement of hemodynamic parameters in cases in which technical success was achieved. Notwithstanding, the rate of observed complications underscores the need to interpret results with care.

This technique may constitute a feasible alternative option in scenarios with limited cath lab access and for patients in whom it is desirable to avoid use of iodinated contrast. Studies with larger numbers of patients and longer follow-up are needed to better determine its safety and durability.

## DISPONIBILIDADE DE DADOS

Todos os dados gerados ou analisados estão incluídos neste artigo e/ou no material suplementar.
