# Supplementary material for: Ultrasound-guided angioplasty of arteriovenous fistula for hemodialysis: initial experience of a single center
Source: J Vasc Bras. 2026 Sep 3;25:e20260022. doi: 10.1590/1677-5449.202600222 (PMC13671020; doi:10.1590/1677-5449.202600222)
Supplement: Translation

# Translation: Angioplastia ecoguiada de fístula arteriovenosa para hemodiálise: experiência inicial de um centro

**DOI:** 10.1590/1677-5449.202600221

**Authors:** Claudia Guimarães Agle, Fábio Luis Barbosa Andrade, Ana Clarice de Carvalho Bacelar, Giovana Pires de Sousa Bonfim, Eduardo Castro de Oliveira, José Siqueira de Araujo

**Affiliations:** 1 Hospital Ana Nery – HAN, Salvador, BA, Brasil.; 2 Complexo Hupes – UFBA, Salvador, BA, Brasil.

**Keywords:** fístula arteriovenosa, angioplastia, ultrassonografia Doppler, hemodiálise, acesso vascular

## Abstract

A doença renal crônica é um problema crescente de saúde pública, e as fístulas arteriovenosas são o acesso de escolha para hemodiálise, embora frequentemente evoluam com estenoses que comprometem sua função. A angioplastia é o tratamento de primeira linha, mas seu acesso é limitado no Sistema Único de Saúde. Este estudo retrospectivo avaliou a factibilidade e os resultados iniciais da angioplastia ecoguiada de fístula arteriovenosa em um centro de referência, em 2024. No total, nove pacientes foram incluídos, com seguimento de 3 meses. Foram avaliados sucesso técnico, fluxo sanguíneo, diâmetro venoso, complicações e perviedade. O sucesso técnico foi de 77,8%, com aumento do fluxo e do diâmetro venoso. Complicações ocorreram em 66,6%, predominando hematomas sem repercussão clínica; dois casos evoluíram com trombose aguda. A técnica mostrou-se factível, com melhora hemodinâmica, embora tenha apresentado uma taxa relevante de complicações.

## INTRODUÇÃO

A doença renal crônica (DRC) representa um problema relevante de saúde pública, com impacto crescente sobre os sistemas de saúde. Dados do Censo Brasileiro de Diálise de 2022 estimam mais de 150 mil pacientes em terapia dialítica no país, com aumento do uso de cateteres venosos centrais e redução do uso de fístulas arteriovenosas (FAVs), evidenciando dificuldades relacionadas à sua confecção, maturação e manutenção^1^.

O acesso vascular ideal para hemodiálise deve oferecer fluxo adequado, baixa taxa de complicações e perviedade duradoura. Nesse contexto, as FAVs permanecem como a melhor opção, sendo associadas a menor morbimortalidade em comparação aos cateteres venosos centrais^2^.

Apesar disso, as FAVs frequentemente evoluem com estenoses venosas secundárias à hiperplasia intimal, comprometendo sua maturação e funcionalidade^3^. Diretrizes nacionais recomendam a realização de ultrassonografia Doppler nos casos de falha de canulação após 12 semanas, visando a identificação precoce de alterações e o planejamento terapêutico^4^.

A angioplastia transluminal percutânea é considerada o tratamento de escolha na maioria desses casos. No entanto, o método convencional depende de infraestrutura de alta complexidade, com uso de contraste iodado e radiação ionizante, o que limita sua disponibilidade no Sistema Único de Saúde. Nesse cenário, a angioplastia ecoguiada surge como uma alternativa promissora, permitindo intervenções com menor custo, sem radiação e sem contraste, sendo especialmente útil em pacientes pré-dialíticos ou com contraindicação ao contraste iodado.

## OBJETIVOS

Primário: avaliar a factibilidade da angioplastia de fístula arteriovenosa guiada por ultrassonografia.

Secundário: descrever variáveis associadas à factibilidade do procedimento, incluindo fluxo sanguíneo pré e pós, complicações e perviedade em curto prazo.

## MÉTODOS

Estudo observacional do tipo coorte retrospectiva, conduzido conforme as diretrizes *Strengthening the Reporting of Observational Studies in Epidemiology* (STROBE)^5^. Foram incluídos todos os pacientes submetidos à angioplastia ecoguiada de FAV no ano de 2024, caracterizando uma amostra de conveniência.

Os critérios de inclusão contemplaram pacientes maiores de 18 anos, com FAV apresentando estenose significativa (> 70%) associada à redução do fluxo (< 600 mL/min) (Figura 1) ou falha de maturação. Não houve critérios de exclusão.

**Figura 1:** Estenose de FAV ao ultrassom Doppler e turbilhonamento de fluxo sanguíneo. FAV = fístula arteriovenosa.

Foram coletados dados de fluxo sanguíneo e diâmetro venoso antes e após o procedimento (Figuras 2 e 3), além de complicações (Figura 4) e avaliação ultrassonográfica após 3 meses.

**Figura 2:** Fluxo sanguíneo em artéria radial (pré-angioplastia) + diâmetro da veia cefálica (pré-angioplastia).

**Figura 3:** Fluxo sanguíneo em artéria radial (pós-angioplastia) + diâmetro da veia cefálica (pós-angioplastia).

**Figura 4:** Hematoma pós angioplastia visualizado ao ultrassom Doppler.

O sucesso técnico foi definido como restauração do fluxo sanguíneo e recanalização da estenose com patência imediata ao final do procedimento. A perviedade foi definida como manutenção da funcionalidade da FAV durante o seguimento de 3 meses, sem necessidade de nova intervenção ou evidência de trombose ao Doppler.

O projeto de pesquisa foi submetido ao Comitê de Ética em Pesquisa do Hospital Ana Nery (HAN), em Salvador, Bahia, e inserido na Plataforma Brasil (Certificado de Apresentação para Apreciação Ética: 83561224.1.0000.0045, com nº do Parecer Consubstanciado: 7.511.150).

### Análise estatística

Foi realizada análise estatística dos dados, expressos em média e desvio-padrão. Para comparação entre valores pré e pós-procedimento, utilizou-se o teste não paramétrico de Wilcoxon para amostras pareadas, considerando um nível de significância de 5%. A análise teve caráter exploratório, em virtude do pequeno tamanho amostral.

### Técnica e materiais de angioplastia

Os procedimentos foram realizados com ultrassom Doppler. A escolha dos balões seguiu critérios técnicos e disponibilidade institucional.

1. Procedimentos realizados com ultrassom Doppler MindRay; inicialmente, foi realizado mapeamento com ultrassonografia Doppler para identificação do local e grau da estenose.
2. Sedação + bloqueio anestésico.
3. Punção ecoguiada retrógrada de artéria radial para acesso percutâneo com posterior passagem de fio-guia sob orientação ultrassonográfica. Foram utilizados introdutor 5 Fr radial, fio-guia hidrofílico 0,035 x 260 mm e cateter vertebral. Sob visualização ultrassonográfica, o fio-guia avançou através da estenose, acompanhado em tempo real.
4. Um cateter-balão foi posicionado sobre o fio-guia, centrado na área de estenose. A dilatação foi realizada sob controle ultrassonográfico, observando a expansão do balão (Figura 5). Foram utilizados balões Conquest e Mustang de 5 a 7 mm (características e tamanhos apresentados na Tabela 1).

**Figura 5:** Balonamento de estenose visualizado ao ultrassom Doppler.

**Tabela 1:** Características técnicas dos balões utilizados nos procedimentos de angioplastia.

	**Diâmetro/ Comprimento**	**Complacência/pressão**	**Marca**
Paciente 01	5x60 mm	Não complacente	Conquest – BD/Bard
	6x60 mm	Ultra alta pressão	
Paciente 02	5x40 mm	Não complacente	Conquest – BD/Bard
	7x40 mm	Ultra alta pressão	
Paciente 03	5x40 mm	Não complacente	Conquest – BD/Bard
	7x40 mm	Ultra alta pressão	
Paciente 04	6x60 mm	Não complacente	Conquest – BD/Bard
		Ultra alta pressão	
Paciente 05	5x80 mm	Não complacente	Conquest – BD/Bard
	7x80 mm	Ultra alta pressão	
Paciente 06	Trombose durante cateterização	-	-
Paciente 07	5x40 mm	Semicomplacente	Mustang - Boston
	6x40 mm	Alta pressão	
Paciente 08	6x60 mm	Semicomplacente	Mustang - Boston
	7x60 mm	Alta pressão	
Paciente 09	6x40 mm	Semicomplacente	Mustang - Boston
	7x40 mm	Alta pressão
5. Após a dilatação, o ultrassom foi utilizado para verificar a resolução da estenose e o aumento do fluxo, bem como a ausência de complicações imediatas.
6. Realizou-se a retirada de introdutor e a compressão local.

## RESULTADOS

Foram realizados nove procedimentos de angioplastia ecoguiada de FAV, com suas respectivas variáveis avaliadas (diâmetro e fluxo sanguíneo) (Tabelas 2, 3 e 4). O sucesso técnico foi alcançado em sete casos (77,8%), enquanto dois pacientes (22,2%) evoluíram com trombose aguda da fístula durante o procedimento, caracterizando falha terapêutica.

**Tabela 2:** Características dos pacientes submetidos à angioplastia ecoguiada de FAV (n = 9).

**Variável**	**Resultado**
Sexo masculino, n (%)	6 (66,7)
Sexo feminino, n (%)	3 (33,3)
FAV radiocefálica, n (%)	6 (66,7)
FAV braquiocefálica, n (%)	3 (33,3)
Dialíticos, n (%)	6 (66,7)
Pré-dialíticos, n (%)	3 (33,3)
Sucesso técnico, n (%)	7 (77,8)
Falha técnica (oclusão), n (%)	2 (22,2)
Tipo de balão, n (%)	
Não complacente (Conquest)	6 (66,7)
Semicomplacente (Mustang)	3 (33,3)

FAV = fístula arteriovenosa.

**Tabela 3:** Fluxo sanguíneo da FAV antes e após angioplastia ecoguiada (ml/min).

	**Fluxo sanguíneo FAV pré**	**Fluxo sanguíneo FAV pós**	**Tipo FAV**
Paciente 01	70,0 ml/min	478,0 ml/min	Radiocefálica
Paciente 02	300,0 ml/min	850,0 ml/min	Radiocefálica
Paciente 03	234,0 ml/min	Oclusão aguda da FAV	Radiocefálica
Paciente 04	190,0 ml/min	700,0 ml/min	Radiocefálica
Paciente 05	152,0 ml/min	787,0 ml/min	Radiocefálica
Paciente 06	253,4 ml/min	Oclusão aguda da FAV	Braquiocefálica
Paciente 07	344,9 ml/min	507,1 ml/min	Radiocefálica
Paciente 08	177,8 ml/min	515,9 ml/min	Braquiocefálica
Paciente 09	346,6 ml/min	500,0 ml/min	Braquiocefálica

FAV = fístula arteriovenosa.

**Tabela 4:** Diâmetro da veia da FAV antes e após angioplastia ecoguiada (mm).

	**Diâmetro da veia pré**	**Diâmetro da veia pós**
Paciente 01	2,0 mm	6,0 mm
Paciente 02	2,0 mm	6,0 mm
Paciente 03	3,7 mm	Oclusão da FAV
Paciente 04	2,0 mm	5,6 mm
Paciente 05	2,8 mm	5,8 mm
Paciente 06	2,0 mm	Oclusão da FAV
Paciente 07	3,1 mm	5,5 mm
Paciente 08	4,1 mm	4,3 mm
Paciente 09	4,0 mm	4,0 mm

FAV = fístula arteriovenosa.

Para a análise comparativa dos parâmetros hemodinâmicos, foram considerados apenas os casos com sucesso técnico e dados completos (n = 7), excluindo os pacientes com trombose aguda intraoperatória.

O fluxo sanguíneo médio aumentou de 225,9 ml/min no pré-procedimento para 619,7 ml/min no pós-procedimento (p = 0,018; teste de Wilcoxon). O diâmetro venoso médio aumentou de 2,9 mm para 5,3 mm, também com diferença estatisticamente significativa (p = 0,017) (Tabela 5).

**Tabela 5:** Análise estatística dos parâmetros hemodinâmicos (apenas nos casos com sucesso técnico, n = 7).

**Variável**	**Pré (média ± DP)**	**Pós (média ± DP)**
Fluxo sanguíneo (ml/min)	225,9 ± 107,7	619,7 ± 154,8
Diâmetro da veia (mm)	2,9 ± 0,9	5,3 ± 0,7

DP = desvio-padrão.

As complicações ocorreram em 66,6% dos casos, sendo a maioria composta por hematomas locais (44,4%). Complicações maiores ocorreram em 22,2% dos casos, correspondendo a trombose aguda da fístula (Tabela 6).

**Tabela 6:** Complicações observadas após angioplastia ecoguiada de FAV – n (%).

Hematomas, n (%)	4 (44,4%)
Oclusão aguda de FAV, n (%)	2 (22,2%)

FAV = fístula arteriovenosa.

Na avaliação ultrassonográfica após 3 meses, observou-se manutenção da perviedade em 77,8% dos casos (sete dos nove pacientes), ou seja, 100% dos casos que obtiveram sucesso imediato, com preservação do fluxo e do diâmetro venoso nos casos funcionais (Tabelas 7 e 8; Figura 6).

**Tabela 7:** Avaliação ultrassonográfica da FAV aos 3 meses após angioplastia ecoguiada.

	**Diâmetro da veia 3 meses após**	**Fluxo sanguíneo FAV 3 meses após**
Paciente 01	6,0 mm	850,0 ml/min
Paciente 02	7,0 mm	800,0 ml/min
Paciente 03	Trombose	FAV ocluída
Paciente 04	5,9 mm	498,0 ml/min
Paciente 05	9,1 mm	989,9 ml/min
Paciente 06	Trombose	FAV ocluída
Paciente 07	6,0 mm	1200,0 ml/min
Paciente 08	4,9 mm	560,0 ml/min
Paciente 09	6,0 mm	690,0 ml/min

FAV = fístula arteriovenosa.

**Tabela 8:** Avaliação ultrassonográfica aos 3 meses (casos com sucesso, n = 7).

**Variável**	**Média ± DP**
Fluxo sanguíneo (ml/min)	798,3 ± 238,7
Diâmetro da veia (mm)	6,4 ± 1,3

DP = desvio-padrão. Perviedade em 3 meses: 100% dos casos que obtiveram sucesso imediato (excluindo-se os casos sem sucesso técnico).

**Figura 6:** Fluxograma.

## DISCUSSÃO

A angioplastia de FAVs guiada por ultrassonografia tem sido progressivamente incorporada como alternativa ao tratamento convencional, especialmente em cenários com limitação de recursos. No presente estudo, o procedimento foi tecnicamente factível na maioria dos casos, com taxa de sucesso de 77,8%, associada à melhora significativa dos parâmetros hemodinâmicos.

Nos casos com sucesso técnico, observou-se aumento do fluxo sanguíneo e do diâmetro venoso, parâmetros fundamentais para a funcionalidade da fístula. Esses achados estão em consonância com a literatura, que aponta o fluxo ao Doppler como marcador de sucesso clínico após intervenções em acessos para hemodiálise^6,7^, além de aproximarem os valores dos critérios descritos pelas diretrizes *Kidney Disease Outcomes Quality Initiative* **(**KDOQI)^8^.

O ultrassom Doppler tem papel central na avaliação, no seguimento e na intervenção em FAVs, por se tratar de um método não invasivo, disponível e capaz de fornecer informações anatômicas e funcionais em tempo real^6,7^.

A angioplastia ecoguiada apresenta vantagens relevantes em relação ao método convencional, como a ausência de radiação ionizante e a não utilização de contraste iodado, aspecto particularmente importante em pacientes com doença renal crônica pré-dialítica. Além disso, a possibilidade de realização fora de ambientes de alta complexidade pode ampliar o acesso ao tratamento, especialmente em sistemas de saúde com limitações estruturais^3,6^.

Estudos prévios demonstram resultados favoráveis com a técnica. Abbadie et al.^6^ e Kazandjian et al.^7^ descreveram melhora dos parâmetros hemodinâmicos e adequada perviedade em curto prazo. Mais recentemente, um estudo multicêntrico brasileiro com balões farmacológicos mostrou bons resultados em eficácia e segurança, com perviedade em médio prazo^9^. Embora não tenham sido utilizados balões farmacológicos no presente estudo, esses dados reforçam o papel da angioplastia no manejo das disfunções de FAV.

Por outro lado, os resultados do presente estudo devem ser interpretados à luz das taxas de complicações observadas. Embora a maioria dos eventos tenha sido composta por complicações menores, como hematomas autolimitados, a taxa global foi elevada (66,6%), incluindo trombose aguda da fístula em 22,2% dos casos, resultando em falha do procedimento. Ainda que parte dessas complicações não tenha impactado clinicamente o funcionamento do acesso, a frequência observada não é desprezível e deve ser analisada de forma crítica.

Na literatura, DerDerian et al.^10^ descreveram taxas relevantes de complicações, com predomínio de eventos menos importantes e uma menor incidência de trombose. A elevada taxa observada neste estudo pode estar relacionada ao pequeno tamanho amostral, à heterogeneidade dos casos e à natureza operador-dependente da técnica.

Adicionalmente, deve-se considerar que as estenoses em FAVs apresentam caráter predominantemente fibrótico, frequentemente exigindo o uso de balões de alta pressão para sua dilatação. Esse aspecto pode contribuir para maior risco de lesão vascular durante o procedimento, influenciando a ocorrência de complicações.

Outro ponto relevante refere-se à variabilidade dos materiais utilizados. No presente estudo, diferentes tipos de balões foram empregados conforme a disponibilidade institucional, cenário que reflete a realidade de muitos serviços, mas que pode influenciar os resultados, especialmente em lesões mais complexas.

Apesar dessas limitações, a angioplastia ecoguiada pode representar uma alternativa factível para o tratamento de estenoses e falha de maturação de FAV, especialmente em cenários com restrição de acesso à hemodinâmica, conforme apontado por diretrizes recentes^4,11,12^.

Este estudo apresenta limitações. Trata-se de uma amostra pequena e de conveniência, o que limita o poder estatístico e a generalização dos achados. Além disso, a amostra foi heterogênea, incluindo pacientes dialíticos e não dialíticos, bem como diferentes tipos de FAVs. A variabilidade dos materiais utilizados, decorrente da disponibilidade institucional, e o caráter operador-dependente do procedimento podem ter influenciado os desfechos observados. Por fim, a taxa de complicações, incluindo eventos maiores como trombose aguda da fístula, deve ser interpretada com cautela, especialmente considerando o tamanho amostral reduzido.

## CONCLUSÃO

A angioplastia ecoguiada de FAVs mostrou-se factível em casos selecionados, com melhora dos parâmetros hemodinâmicos nos casos com sucesso técnico. Contudo, a taxa de complicações observada reforça a necessidade de uma interpretação cautelosa.

A técnica pode representar uma alternativa viável em cenários com limitação de acesso à hemodinâmica e em pacientes nos quais se deseja evitar o uso de contraste iodado. Estudos com maior número de pacientes e seguimento mais prolongado são necessários para melhor definição de sua segurança e durabilidade.

## Data Availability

All of the data generated or analyzed are included in the article and/or supplementary material.
